# Deep RNA-Seq reveals miRNome differences in mammary tissue of lactating Holstein and Montbéliarde cows

**DOI:** 10.1186/s12864-019-5987-4

**Published:** 2019-07-30

**Authors:** P. A. Billa, Y. Faulconnier, T. Ye, M. Chervet, F. Le Provost, J. A. A. Pires, C. Leroux

**Affiliations:** 1 Institut National de la Recherche Agronomique (INRA), Université Clermont Auvergne, VetAgro Sup, UMR Herbivores, UMR1213 Herbivores, F-63122 Saint-Genès-Champanelle, France; 2 Institut de Génétique et de Biologie Moléculaire et Cellulaire (IGBMC), Centre National de la Recherche Scientifique, UMR7104, Institut National de la Santé et de la Recherche Médicale, U964, Université de Strasbourg, 67404 Illkirch, France; 3 Department of Food Science & Technology, University of California Davis, Davis, CA, USA; 4 GABI, INRA, AgroParisTech, Université Paris-Saclay, Jouy-en-Josas, F-78352 France

**Keywords:** MiRNome, Mammary gland, Dairy cows, Breeds, RNA-Seq, Differentially expressed

## Abstract

**Background:**

Genetic polymorphisms are known to influence milk production and composition. However, the genomic mechanisms involved in the genetic regulation of milk component synthesis are not completely understood. MicroRNAs (miRNAs) regulate gene expression. Previous research suggests that the high developmental potential of the mammary gland may depend in part on a specific miRNA expression pattern. The objective of the present study was to compare the mammary gland miRNomes of two dairy cow breeds, Holstein and Montbéliarde, which have different mammogenic potentials that are related to differences in dairy performance.

**Results:**

Milk, fat, protein, and lactose yields were lower in Montbéliarde cows than in Holstein cows. We detected 754 distinct miRNAs in the mammary glands of Holstein (*n* = 5) and Montbéliarde (*n* = 6) midlactating cows using RNA-Seq technology, among which 738 were known and 16 were predicted miRNAs. The 25 most abundant miRNAs accounted for 90.6% of the total reads. The comparison of their abundances in the mammary glands of Holstein versus Montbéliarde cows identified 22 differentially expressed miRNAs (P_adj_ ≤ 0.05). Among them, 11 presented a fold change ≥2, and 2 (*miR-100* and *miR-146b*) were highly expressed. Among the most abundant miRNAs, *miR-186* is known to inhibit cell proliferation and epithelial-to-mesenchymal transition.

Data mining showed that 17 differentially expressed miRNAs with more than 20 reads were involved in the regulation of mammary gland plasticity. Several of them may potentially target mRNAs involved in signaling pathways (such as mTOR) and lipid metabolism, thereby indicating that they could influence milk composition.

**Conclusion:**

We found differences in the mammary gland miRNomes of two dairy cattle breeds. These differences suggest a potential role for miRNAs in mammary gland plasticity and milk component synthesis, both of which are related to milk production and composition. Further research is warranted on the genetic regulation of miRNAs and their role in milk synthesis.

**Electronic supplementary material:**

The online version of this article (10.1186/s12864-019-5987-4) contains supplementary material, which is available to authorized users.

## Background

The mammary gland (MG) is a complex secretory organ composed of different cell types that interact to ensure proper mammary function and milk synthesis. The rate of MG development and dairy potential differ among breeds. For instance, milk, fat, and protein yield were greater for Holstein (22.7, 0.8, and 0.7 kg/day, respectively) than for Montbéliarde cows (17.6, 0.6, and 0.5 kg/day, respectively) in mountain grazing conditions [1]. Similarly, a comparison between Holstein and Montbéliarde cows fed a maize silage-based diet showed higher milk, fat, and protein yield in Holstein (35.7, 1.4 and 1.1 kg/day, respectively) than in Montbéliarde cows (28.1, 1.1, and 0.9 kg/day, respectively) [2]. The fat yield differences were accompanied by differences in milk fatty acid composition [2]. Genetic and, more recently, genomic selection of dairy cows has led to increased milk production resulting from the increased secretory capacity of the MG [3]. The mammogenic potential in dairy heifers in comparison with beef heifers is facilitated by an increased number of mammary stem cells and the differential expression of genes involved in the development of the mammary stem cell niche. These genes influence the proliferation, migration, differentiation, and remodeling of mammary tissue and the regulation of adipocyte transdifferentiation [4]. As a result, genetic polymorphisms that differentiate species and breeds also influence milk production and composition. Despite the increase in knowledge about the genetics of dairy cows in recent decades, the genomic mechanisms influencing milk secretion and composition are not fully understood.

MicroRNAs (miRNAs) are small noncoding RNAs with 18–25 nucleotides. They regulate gene expression by base-pairing with mRNA to induce their degradation or to inhibit their translation [5]. MiRNAs are thought to regulate at least 60% of genes. Therefore, they are involved in many different cellular processes [6–9]. Nevertheless, the genetic regulation of miRNAs is poorly understood. A recent study identified 125 differentially expressed miRNAs in the kidney among 3 distinct porcine breeds. This suggests that miRNAs could be used for the study of the genetic variability underlying complex traits [10]. Similarly, the identification of 50 miRNAs that were differentially abundant in serum from Warmblood horses (Arabian, Anglo-Arabian, Selle Français, Cob Normand, French trotter and Trait du Nord breeds) and ponies (Shetland and Welsh breeds) suggests the potential of miRNA to serve as biomarkers of different equine breeds [11]. In cows, the miRNomes of ruminant MGs were investigated using RNA-Seq, which is a powerful tool to characterize a large miRNA repertoire [12–16]. The roles of miRNAs during mammary development have also been reported [17–19]. While the physiological [15] and nutritional [20–22] regulation of MG miRNomes in bovine and caprine species have been reported, the genetic influence on miRNA gene expression is still limited. A comparison of MGs from different bovine breeds with contrasted mammogenic potential found 54 differentially expressed miRNAs (DEMs) in mammary tissues in dairy (Holstein-Friesian) and beef (Limousin) postpubertal heifers. These results suggest that the high developmental potential of the MG in dairy cattle, leading to high milk production, may depend on the specific miRNA expression pattern [23]. Thus, the objective of the present study was to compare MG miRNomes in Holstein and Montbéliarde cows, which are two dairy breeds with different dairy performances.

## Results & discussion

### Milk production and composition

**Fig. 1:**
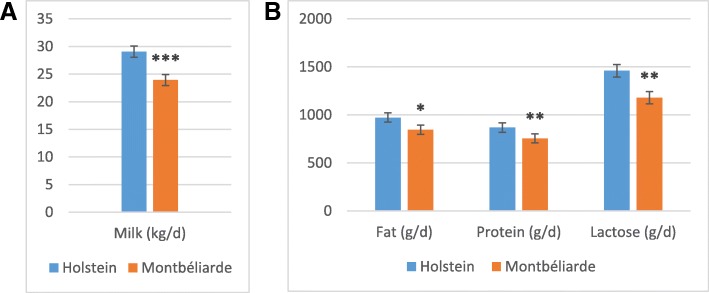
Milk production (**a**) and composition (**b**). A comparison between Holstein (*n* = 5) lactating cows and Montbéliarde (*n* = 6) lactating cows. *** *P* < 0.001. ** *P* < 0.01. * *P* < 0.05. Bars are SEM

Milk production and composition were measured before mammary biopsies to characterize the performance of cows in the present study. The milk yield was higher in Holstein than in Montbéliarde cows (29.1 vs 23.9 kg/day; Fig. 1a). Fat, protein and lactose yields were higher in Holstein (972, 869, and 1460 g/day, respectively) than in Montbéliarde cows (846, 756, and 1179 g/day, respectively; Fig. 1b). These differences are in agreement with those found in previous studies comparing lactating Holstein and Montbéliarde cows fed diets based on maize silage [2] and grazed grass [1].

### Global description of mammary miRNomes

**Fig. 2:**
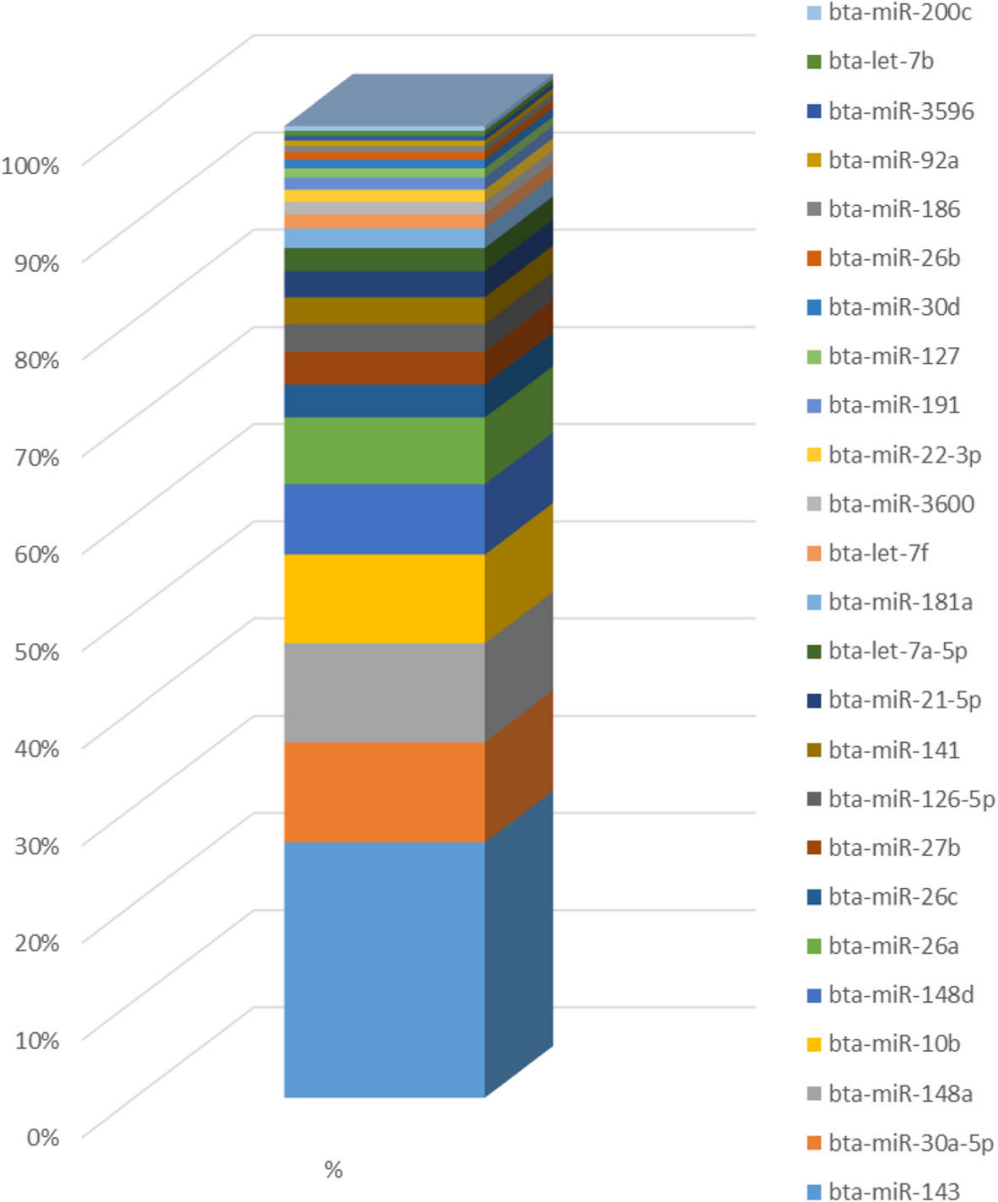
Top 25 most abundant miRNAs in the mammary gland of lactating cows. Representation of the percentage of reads of each miRNA relative to the total reads of the Holstein (*n* = 5) libraries and Montbéliarde (*n* = 6) libraries

Five and six libraries were constructed using RNA extracted from the MG of lactating Holstein and Montbéliarde cows, respectively. The high-throughput sequencing performed allowed us to obtain more than 12 million raw reads on average (Table 1). After cleaning (poly-A stretches and adaptors were removed), 11.0 and 8.5 million cleaned reads were mapped onto the *Bos taurus* genome from the Holstein and Montbéliarde libraries, respectively. The percentage of total mapped reads ranged from 96.1 to 98.3% and was comparable between the libraries (Additional file 1: Figure S1). The correlation between the libraries that was calculated using the log_2_ of the normalized counts of expressed miRNAs was R = 0.96, indicating a strong correlation between the Holstein and Montbéliarde libraries (Additional file 1: Figure S2). A total of 754 miRNAs were identified using miRDeep2 software, 738 of which were known and 16 were predicted miRNAs. The latter may be considered to be potentially novel miRNAs. These results are in accordance with data previously obtained using NGS technology in bovine MG that reported 487 known bovine miRNAs, 167 of which were miRNAs that were already known in other species [14]. However, the number of predicted novel miRNAs was higher in the study by Le Guillou et al. [14] than in our study (679 vs 16, respectively). In the present study, we used the latest miRBase version, which includes a large number of known miRNAs, and this could explain the lower number of predicted miRNAs. The 25 most abundant miRNAs in the MG represented 90.6% of the total reads. Among them, four (*miR-143*, *miR-30a-5p*, *miR-148a*, and *miR-10b*) represented more than 50% of the total reads (Fig. 2).

**Table 1:** Sequencing data from Holstein (*n* = 5) and Montbéliarde (*n* = 6) lactating cows. Clean reads were after size (15 to 40 nt), adapter and soft-clipping cleaning

Breed	Raw reads	Too short	Adapter	Cleaned reads	Output rate(%)
Holstein	13393626.2	724537.6	56192.0	11027924.4	82.3
Montbéliarde	11953902.2	1405670.2	824394.2	8500864.8	71.1

### Differentially expressed miRNAs in the mammary gland of Holstein and Montbéliarde cows

**Fig. 3:**
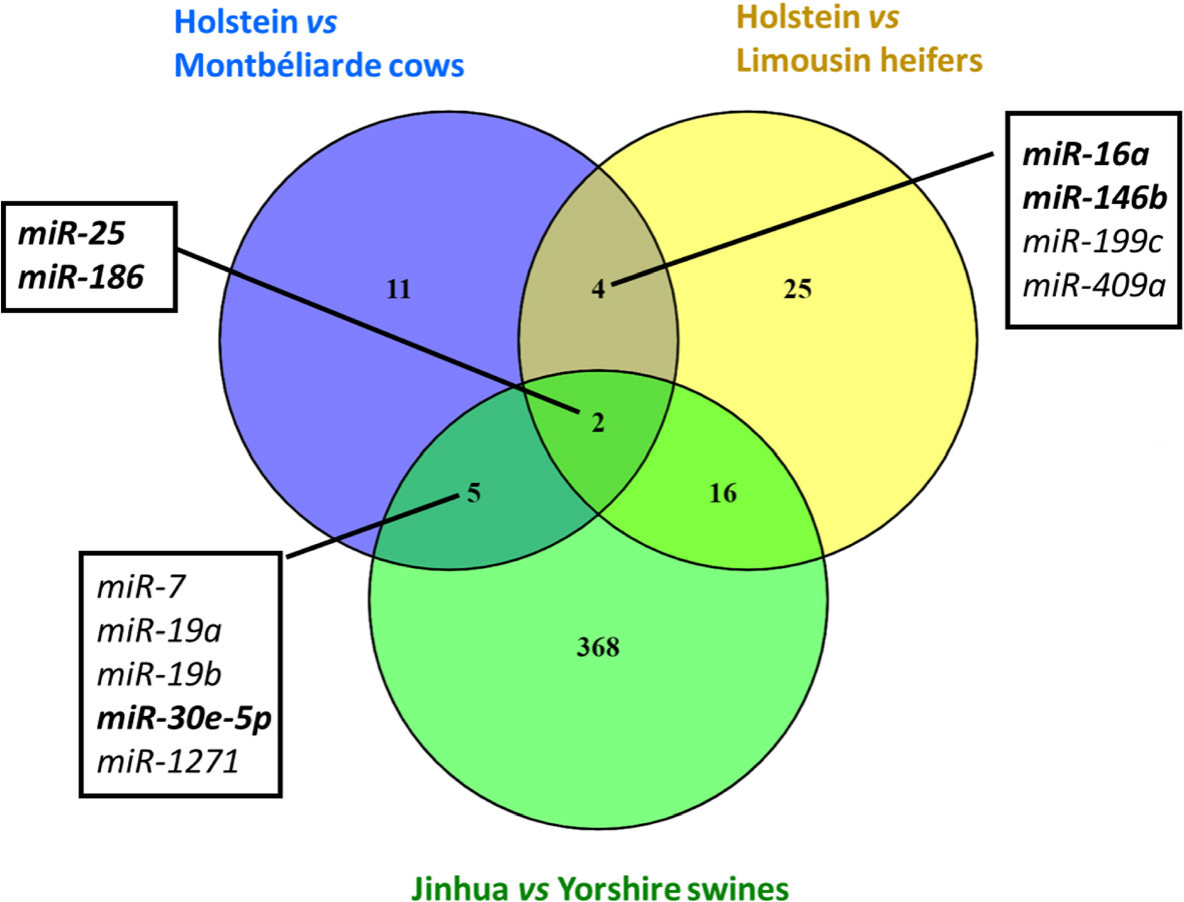
Venn diagram of DEMs in different mammary gland studies. The list of the comparison between Jinhua and Yorshine swine and between Holstein and Limousin heifers were from Peng et al. [23] and Wicik et al. [24], respectively. The name of the common miRNAs with the present study was indicated. In bold there were those highly expressed

A comparison of the MG miRNomes of Holstein and Montbéliarde cows using the DESeq2 package allowed the identification of 22 miRNAs that were differentially expressed (P_adj_ ≤ 0.05; Table 2) between the breeds. Among them, 11 were up- and 11 were down-regulated in Holstein cows compared to Montbéliarde cows, and 17 were found to correspond to more than 20 reads on average. Six (*miR-16a*, *miR-186*, *miR-25*, *miR-100*, *mir-30e-5p* and *miR-146b*) corresponded to more than 5,000 reads on average. Among these 22 DEMs, 11 presented a fold change ≥2, and *miR-100* and *miR-146b* were highly expressed miRNAs. Breed-specific patterns in miRNomes have also been observed in porcine kidney [10], equine serum [11], and porcine and bovine MG [23, 24]. In porcine MG, a miRNome comparison between the Jinhua and Yorkshire breeds identified 391 DEMs [24]. This higher number of DEMs might be due to the large differences in the numbers of predicted miRNAs. Indeed, the stringency of our analysis allowed us to detect only 16 predicted miRNAs, whereas the use of a BLAST strategy identified 2823 and 2286 potential miRNAs in Jinhua and Yorkshire breeds, respectively. In addition, the breed comparison in the porcine study showed large differences in terms of genetic selection and therefore in lactation performance. The Jinhua is a traditional breed that produces less milk than the selected Yorkshire breed. The large divergence in phenotype and the difference in the predicted number of miRNAs could in part explain the detection of a higher number of DEMs in the porcine study. Nevertheless, five DEMs (*miR-7*, *miR-19a*, *miR-19b*, *miR-30e-5p*, and *miR-1271*) were identified in both the present and the porcine study, including *miR-30e-5p,* which was highly expressed in MG in cows (Fig. 3). In bovine MG, Wicik et al. [23] identified 54 DEMs in beef and dairy heifer miRNome comparisons. In the present study, Holstein and Montbéliarde cows have closer milk performance than Holstein and beef Limousin cattle compared by Wicik et al. [23]. Therefore, it is not surprising that the MG miRNomes were more similar in Holstein and Montbéliarde cows than when comparing beef and dairy breeds. Among the 22 DEMs identified in the present study, six DEMs (*miR-16a*, *miR-186*, *miR-25*, *miR-409a*, *mir-199c* and *miR-146b*) were among the 54 DEMs detected by Wicik et al. [23] (Fig. 3), and four (*miR-16a*, *miR-186*, *miR-25*, and *miR-146b*) were highly expressed in our study. The comparison between the DEMs found in the studies on the effects of breed on the MG miRNomes in husbandry animals (from present study, Peng et al. [24] and Wicik et al. [23]) revealed two miRNAs, *miR-25* and *miR-186,* that are shared. Therefore, these two miRNAs are of interest as their expression could be related to the selection of genes involved in milk production.

**Table 2:** Differentially expressed miRNAs. MiRNAs whose expression was different in lactating mammary glands of Holstein (H) and Montbéliarde (M) cows. ^a^for *n* = 5 and ^b^for *n* = 6. MiRNAs with higher abundance in Holstein cows are in italic and miRNAs with more than 20 reads on average are in bold. Means correspond to the mean of normalized read counts

	Mean reads		log_2_FC	FDR
	H^a^	M^b^	(M/H)	
*bta-miR-7*	***193.31***	***107.92***	***−0.85***	***9.33E-04***
*bta-miR-16a*	***14054.01***	***8193.46***	***−0.78***	***1.16E-04***
*bta-miR-19a*	***144.51***	***85.90***	***−0.75***	***3.64E-02***
*bta-miR-19b*	***1641.70***	***1085.81***	***−0.59***	***1.88E-02***
bta-miR-25	**15748.75**	**20362.65**	**0.37**	**2.20E-02**
*bta-miR-30e-5p*	***39192.39***	***30598.24***	***−0.36***	***3.64E-02***
bta-miR-100	**5606.75**	**11221.56**	**1.00**	**3.64E-02**
bta-miR-146b	**4687.51**	**14263.31**	**1.61**	**4.24E-02**
*bta-miR-186*	***71355.31***	***42157.25***	***−0.76***	***3.92E-04***
bta-miR-199c	**43.22**	**79.99**	**0.88**	**4.27E-02**
*bta-miR-362-5p*	***139.50***	***75.42***	***−0.88***	***3.80E-03***
bta-miR-409a	**8.44**	**31.57**	**1.93**	**4.35E-03**
bta-miR-455-5p	**95.95**	**177.76**	**0.88**	**3.07E-02**
bta-miR-504	**74.54**	**168.71**	**1.18**	**4.00E-02**
*bta-miR-11978*	*23.63*	*10.28*	*−1.23*	*5.11E-02*
bta-miR-1271	**75.38**	**163.79**	**1.12**	**3.64E-02**
bta-miR-1388-3p	**105.46**	**233.08**	**1.14**	**4.32E-03**
*bta-miR-1911*	*20.40*	*2.07*	*−3.18*	*3.64E-02*
*bta-miR-2285ba*	***40.26***	***20.70***	***−0.93***	***3.64E-02***
*bta-miR-2419-3p*	*10.19*	*0.72*	*−4.11*	*3.64E-02*
bta-miR-6523b	**14.39**	**63.09**	**2.12**	**4.78E-02**
chr21_22372_star	232.16	524.19	1.17	3.64E-02

### Putative functions of the 17 differentially expressed miRNAs

Bioinformatic analyses were performed to identify the biological processes potentially regulated by the 17 DEMs with more than 20 reads (on average) and one predicted miRNA (chr21_22372_star), which was not used for the functional analysis. Among the first 20 identified process networks, 12 were linked to development, the regulation of the cytoskeleton, the establishment and regulation of tissues, the cell cycle, and apoptosis (Table 3). These results suggest a role played by the 17 DEMs in the morphology and functioning of mammary tissue. In particular, one process network was linked to epithelial-to-mesenchymal transition (EMT), which has already been reported to be important for MG development and remodeling [25]. Indeed, the polarization of mammary tissue architecture is crucial for the maintenance of transcription factor activation, chromatin organization, and tissue-specific gene expression, which influence milk synthesis [26].

**Table 3:** Biological processes potentially regulated by the 17 DEMs. The 17 miRNAs were selected as having more than a mean of 20 reads**.** Process Networks analysis using Metacore™ software. * indicate processes linked with cytoskeleton and cell life and § indicate signal transduction processes. FDR: False Discovery Rate

	#	Networks	FDR
§	1	Signal transduction NOTCH signaling	8.56E-07
*	2	Cytoskeleton regulation of cytoskeleton rearrangement	2.17E-05
*	3	Development blood vessel morphogenesis	2.57E-05
§	4	Signal transduction WNT signaling	2.92E-04
*	5	Development regulation of angiogenesis	2.92E-04
	6	Development neurogenesis axonal guidance	2.92E-04
	7	Immune response TCR signaling	2.93E-04
*	8	Cell cycle G1-S growth factor regulation	3.48E-04
*	9	Apoptosis anti-apoptosis mediated by external signals via NF-kB	3.48E-04
*	10	Cytoskeleton actin filament	6.87E-04
*	11	Proliferation lymphocyte proliferation	9.41E-04
	12	Inflammation Protein C signaling	1.05E-03
*	13	Development EMT regulation of epithelial-to-mesenchymal transition	1.09E-03
*	14	Cell adhesion attractive and repulsive receptors	1.33E-03
	15	Cardiac development FGF ErbB signaling	1.36E-03
*	16	Proliferation positive regulation cell proliferation	1.36E-03
*	17	Cell cycle G2-M	1.36E-03
	18	Development Hedgehog signaling	1.60E-03
*	19	Cell cycle G1-S interleukin regulation	1.68E-03
	20	Reproduction FSH-beta signaling pathway	1.68E-03

The first modified process was NOTCH signaling transduction. This signaling pathway is involved in cell differentiation and influences mammary development by promoting mammary epithelial stem cell activity [27]. The second identified signal transduction process was Wnt signaling. Wnt proteins influence the self-renewal of stem cells in the MG [28], and recent data indicate that Wnt signaling is also involved in mammary stem cell maintenance by generating MG plasticity in mammary lineage cells [29]. The interactions between epithelial cells and adipocytes coordinate MG development and influence milk production [30]. During lactation, cell turnover was estimated to be ~ 50% due to cell proliferation and apoptosis [3], thereby showing the importance of cell life during lactation. More recently, two mammary epithelial cell populations were linked to milk production, showing the importance of these types of cells [31]. These previously reported data reinforce the importance of MG plasticity for efficient MG development and lactation. Our results based on algorithmic prediction suggest that miRNAs may influence the cell life cycle, cell differentiation and transdifferentiation (e.g., EMT). The occurrence of these mechanisms in MG are still poorly documented and need further research.

### Role of the 6 most differentially expressed miRNAs

**Fig. 4:**
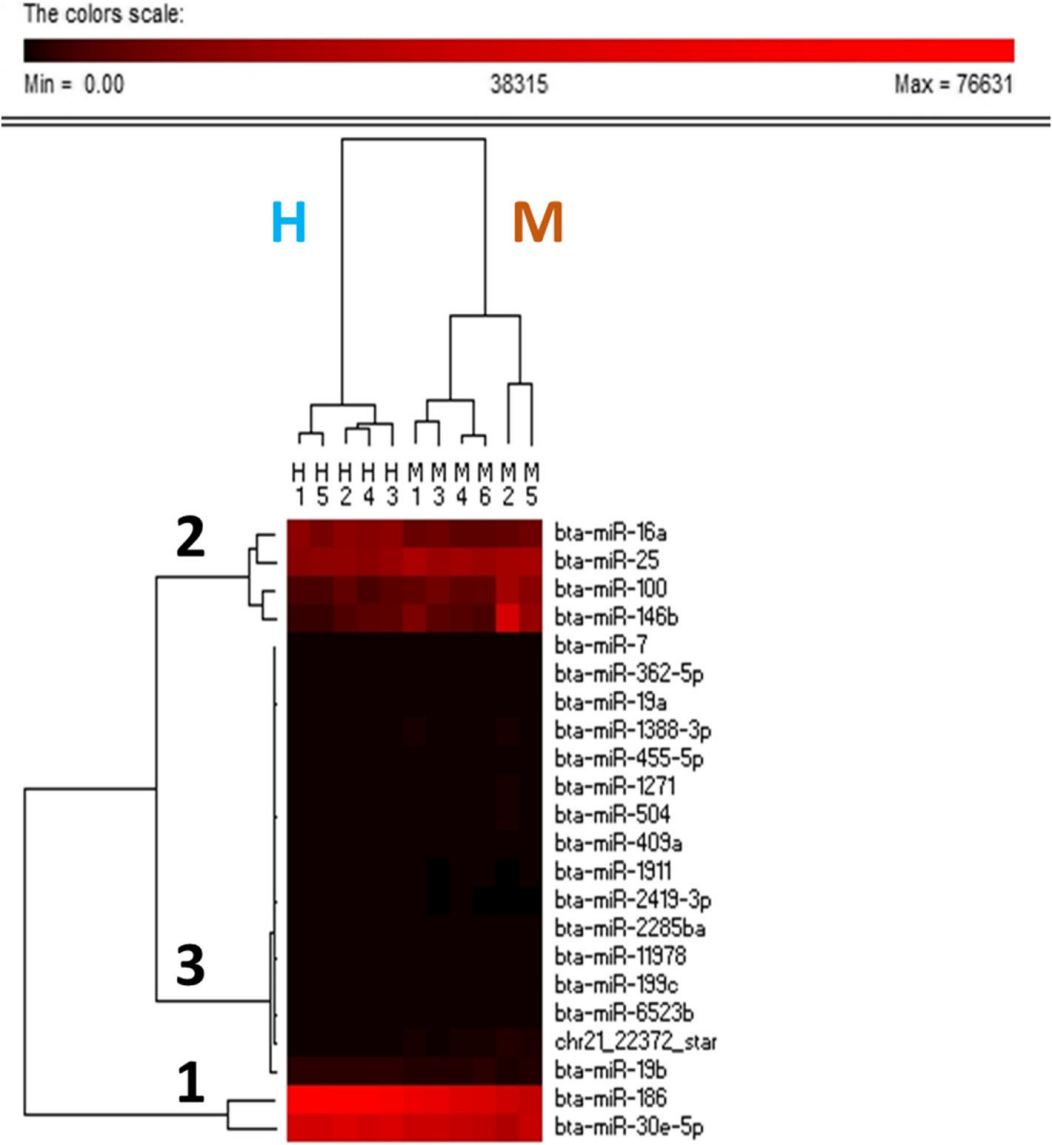
Two-way hierarchical clustering dendrogram of differentially expressed miRNAs. Clustering performed with data of the 11 mammary samples of the 19 DEMS (P_adj_ < 0.05), and using PermutMatrix software (Caraux and Pinloche, 2005) using Euclidean distance calculation and Ward’s minimum variance method. Columns represent each mammary sample for Holstein (H, samples 1 to 5) and Montbéliarde (M, samples 1 to 6) cows. Rows represent each miRNA grouped according to abundance (from Group 1 with higher to Group 3 with lesser abundance)

The hierarchical classification of DEMs using PermutMatrix software allowed the classification of cows according to their breed and the clustering of miRNAs according to their level of expression (Fig. 4). Three groups were identified. The first group corresponded to *miR-186* and *miR-30e-5p,* both of which showed a high expression level. The second group comprised *miR-25, miR-16a, miR-100*, and *miR-146b,* which showed an intermediate expression level. The third group comprised DEMs with low expression. Only the first and second groups (containing 6 miRNAs: *miR-100*, *miR-146b*, *miR-186, miR-30e-5p, miR-25,* and *miR-16a*) were considered for further investigation and discussion. RT-qPCR analyses were performed for *miR-25, miR-146b, miR-100,* and *miR-186*, and *miR-25*, *miR*-*146b*, and *miR*-*186* showed the same expression pattern as that obtained using RNA-Seq (Additional file 1: Table S1).

#### Putative roles of miR-100 and miR-146b, the two most highly expressed miRNAs with fold-changes > 2 in the mammary gland

*MiR-100* and *miR-146b* exhibited fold-changes (FC) > 2 and mean counts > 8,000. *MiR-146b* is involved in the control of the immune system [32] by suppressing the expression of inflammatory cytokines and inducing the inhibition of autophagy by targeting the PTEN/Akt/mTOR signaling pathway [33]. *MiR-100* is identified in numerous cancers [34–36] and acts via mTOR signaling in human mesenchymal stem cells [37]. Elsewhere, mTOR signaling regulates cell growth, proliferation, and cell life, as well as gene transcription and protein synthesis in the MG and could be related to lactation [38, 39]. In addition, mTOR was reported to affect lipogenic gene networks in bovine mammary epithelial cells, indicating a potentially important role in the regulation of milk fat synthesis [40]. The potential regulation of mTOR signaling by *miR-100* and *miR-146b* and their differential expression in the present study could be related to the differences in milk fat and protein yield between Holstein and Montbéliarde cows. However, this indirect relationship has to be studied further. In addition, *miR-100* is reported to induce EMT in a model of mammary epithelial cells, which is in line with the identified biological processes (Table 3) and mammosphere formation [41].

#### Putative roles of the four DEMs with high expression: *miR-186*, *miR-30e-5p*, *miR-25* and *miR-16a*

*MiR-18*6*, miR-30e-5p,* and *miR-16a* were more abundant and *miR-25 was* less abundant in Holstein cows than in Montbéliarde cows. In particular, *miR-186* was one of the 25 most abundant miRNAs in MG tissue (Table 2). *MiR-186* inhibits cell proliferation in prostate [42], gastric [43], melanoma [44], and colorectal [45] cancers. In contrast, it has been reported that *miR-186* promotes cell proliferation in human melanoma [46]. The mechanism of its action is still unclear, but *miR-186* seems to influence cell proliferation. In addition, the downregulation of *miR-186* expression was associated with the EMT phenotype in cisplatin-resistant ovarian cancer [47] and colorectal cancer [45]. *MiR-186* was also reported to regulate glucose uptake and lactate production by targeting the 3′-UTR of *glucose transporter 1* (*Glut1*) mRNA in cancer-associated fibroblasts [48]. Glucose plays a key role in milk synthesis as a precursor for the synthesis of lactose, which is a major driver of milk volume [49]. However, the upregulation of *miR-186* in the MG in Holstein cows is not consistent with the higher lactose yield observed in Holstein cows. Thus, the potential role of *miR-186* in the regulation of lactose synthesis and milk yield needs further investigation.

*MiR-30e-5p* was the second most abundant miRNAs among the DEMs and showed a higher abundance in the MG in Holstein cows. The expression of *miR-30e-5p* in goat mammary epithelial cells was associated with the upregulation of the expression of genes involved in lipid metabolism, such as *PPARγ*, *LPL*, *DGAT1*, and *CD36*, to promote milk fat synthesis [50].

*MiR-25* was the third most highly expressed miRNAs among the DEMs and had greater expression in Montbéliarde cows than in Holstein cows. This miRNA represses triacylglycerol synthesis and lipid accumulation in goat mammary epithelial cells [51]. Indeed, *miR-25* directly targets peroxisome proliferative activated receptor gamma coactivator 1 beta (*PGC-1beta*) and reduces the mRNA levels of *SREBP1*, *FASN*, *GPAM* and *PPARG* [51]. The expression of *miR-16a* was lower in Montbéliarde cows than in Holstein cows. *MiR-16a* was reported to target genes involved in lipid metabolism [52].

#### Links between milk production and composition and DEMs

Bibliographic analyses indicated that the EMT process, which could be related to the structure of mammary epithelium-synthetizing milk components, could be modulated by highly expressed DEMs. Elsewhere, genes involved in the mTOR signaling pathway, reported to be related to protein and lipid synthesis [38–40], were predicted to be targeted by highly expressed DEMs. Lipid metabolism was previously shown to be regulated by *miR-30e-5p*, *miR-25* and *miR-16a*. Therefore, we can suggest a potential role for the highly expressed DEMs in milk component synthesis.

Furthermore, Spearman correlation analysis was performed showing significant correlations between *miR-186, miR-30e-5p,* and *miR-16a* expression and milk, protein, fat and lactose secretion yields (Additional file 1: Table S2; *P* ≤ 0.05). These results supported the hypothesis regarding the potential role of several DEMs in milk production and composition. However, the direct role of these miRNAs in milk synthesis needs to be confirmed by functional analyses, which would provide compelling evidence of their role.

## Conclusion

**Fig. 5:**
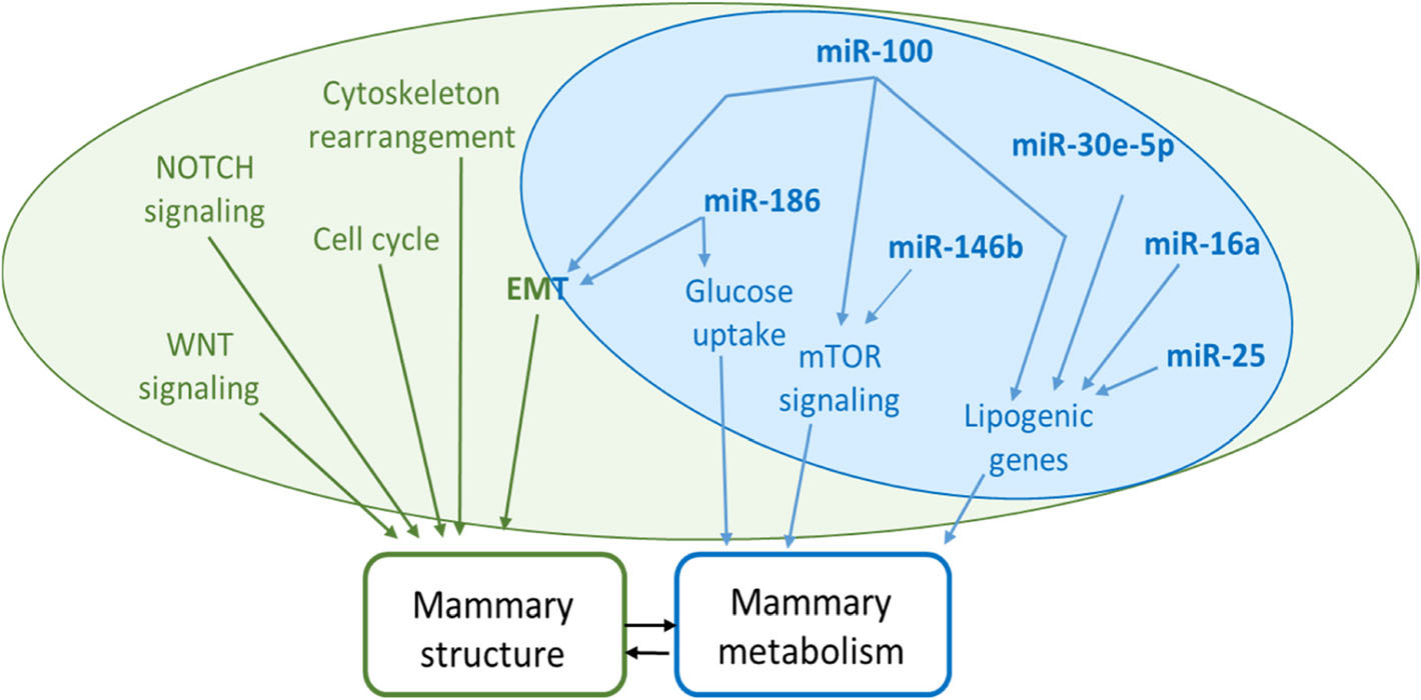
Biological processes and genes potentially modulated. Biological processes potentially regulated (P_adj_ ≤ 0.05) by the 19 DEMS and genes potentially targeted by the 6 most expressed and/or differentially expressed miRNAs. The modified process networks are represented in green and the selected miRNA targeting genes are represented in blue

We found 22 DEMs by comparing the MG miRNomes between two dairy cattle breeds with different dairy performance (Holstein cows and Montbéliarde cows). The bioinformatic analyses of their predicted target genes identified genes involved in signaling networks and tissue structure. We identified 6 miRNAs (*miR-100*, *miR-146b*, *miR-186, miR-30e-5p, miR-25,* and *miR-16a*) which were highly expressed in lactating MG in both breeds. These microRNAs are predicted to target genes involved in the synthesis of milk constituents. In addition, several DEMs are predicted to target the mTOR signaling pathway and genes involved in lipid metabolism, which could be related to the lower milk fat yield in Montbéliarde cows compared to that in Holstein cows (Fig. 5). Among the 6 highly expressed DEMs, we found a correlation between 3 DEMs and milk production and composition supports this hypothesis. Nonetheless, further research is warranted on the genetic regulation of miRNAs and their precise functional roles.

## Methods

### Animals and sampling

Animal procedures were performed in compliance with Regional Animal Care Committee guidelines CEMEAA: Auvergne, French Ministry of Agriculture and European Union guidelines for animal research C2EA-02. All procedures were approved by the ethics committee on animal experimentation (3737-2015043014541577 V2).

Twelve cows (6 Holstein and 6 Montbéliarde), with an average of 3.3 ± 1.5 as lactation number were studied at mid-lactation (165 ± 21 days in milk (DIM)). However, one Holstein cow was excluded from the experiment due to mastitis. The diet was the same for all animals and was composed of forage and concentrate (74:26 of dry matter). Milk yield and composition were recorded on 9 consecutive days before biopsies. The milk was analyzed via mid-infrared spectroscopy to determine the fat and protein content (Galilait, Theix, France).

Mammary biopsies were performed as previously described [53]. The biopsy site was selected at a midpoint on a rear quarter. The collected tissue, approximately 600–650 mg, was rinsed in sterile 0.9% saline solution, inspected to verify tissue homogeneity, and snap-frozen in liquid nitrogen and stored at − 80 °C until RNA extraction.

### RNA preparation and RNA-Seq analysis

Total RNA was extracted from on average 50 mg MG biopsies (*n* = 5 Holstein and *n* = 6 Montbéliarde) using miRVana kit (Thermo Fisher Sciences, USA). The concentration and purity of the RNA was estimated by spectrophotometry NanodropTH (ND-1000, NanoDrop Technologies LLC, Wilmington, DE, USA) and using the Bioanalyzer 2100 (Agilent Technologies Inc., Santa Clara, CA, USA), respectively. Means of RIN were 8.0 and 7.8 in Holstein and Montbéliarde, respectively. The preparation of the libraries were performed by the IGBMC Microarray and Sequencing Platform (Strasbourg, France). Small RNA-Seq librairies were generated from 2000 ng of total RNA using TruSeq Small RNA Library Prep Kit (Illumina, San Diego, CA), according to manufacturer’s instructions. The protocol takes advantage of the natural structure common to most microRNA molecules that have a 3′ hydroxyl group resulting from enzymatic cleavage by Dicer or other RNA processing enzymes. Briefly, during the first step, RNA adapters were sequentially ligated to each end of the RNA; firstly the 3′ RNA adapter (5′ TGGAATTCTCGGGTGCCAAGG 3′) which is specifically designed to target microRNAs and other small RNAs, then the 5′ RNA adapter (5′ GTTCAGAGTTCTACAGTCCGACGATC 3′). Small RNA ligated with 3′ and 5′ RNA adapters were reverse transcribed and PCR amplified (30 s at 98 °C; [10 s at 98 °C, 30 s at 60 °C, 15 s at 72 °C] × 13 cycles; 10 min at 72 °C) to obtain cDNA. Acrylamide gel purifications of 140–160 nt amplified cDNA (corresponding to cDNA obtained from small RNA + 120 nt from the adapters) were performed. The final cDNA libraries were checked for quality and quantified using capillary electrophoresis. Libraries were loaded in the flowcell at 2.8 nM and clusters were generated using Cbot and sequenced on HiSeq 4000 (Illumina) as single-end 50 base reads, according to the manufacturer’s instructions. The quantity and quality of reads for each library are shown in Additional file 1: Table S1. After trimming of adaptor sequences and removal of reads containing ambiguous base calls (FASTX-Toolkit, http://hannonlab.cshl.edu/fastx_toolkit/index.html), reads were filtered according to their size (15–40 nt). Reads quantification and annotation were performed using the ncPRO-seq pipeline [54]. The sequence reads were aligned against the *Bos taurus* btau5.0.1 genome as miRBase_v22.1 using the miRDeep2 package [55]. Precursors and mature miRNAs were identified using the miRDeep2 core module, miRDeep2.pl. Potential miRNAs were annotated accordingly against ortholog miRNAs in goat, sheep and humans (miRBase release 22.1). We used a miRDeep2 score ≥ 0 as a cut-off threshold. The accession number of the RNA-Seq data is GSE131057. The X- (Holstein) and Y- (Montbéliarde) axes show the Log_2_ of the mean of the normalized counts of expressed miRNAs (Additional file 1: Figure S2).

### RT-qPCR analyses

RT-qPCR were performed to confirm the expression pattern, using TaqManTM Advanced miRNA cDNA synthesis and TaqManTM Advanced Master Mix (Life Technologies, Foster City, CA, USA), according to the manufacturer’s instructions. Reverse-transcriptions were achieved on 10 ng of total RNA. PCR were performed using StepOnePlus (Applied Biosystems, Foster City, CA, USA) at 95 °C for 20 s, pursued by 40 cycles of 95 °C for 1 s and 60 °C for 20 s. Used amplification systems (*miR-100*, *-25*, *-146b*, *-186*, *-26a*, *-92a*, and *-191*) are presented in Additional file 1: Table S1. All miRNA levels were normalized to the mean of the values of 3 internal control miRNAs (*miR-26a* [56], *-92a* [57], and *-191* [58]). These miRNAs were identified as suitable internal controls using geNorm (Additional file 1: Figure S3). The results were expressed as log_2_ of fold changes of threshold cycle (Ct) values relative to the control [59].

### Statistical and bioinformatics analyses

Statistical analyses to compare breed effects on the milk production means per cow were performed using mixed models of SAS (version 9.4; SAS Institute INC, Cary, NC). The normalization and differential expression analysis of the known and predicted miRNAs were conducted with the DESeq2 R package v1.18.1. Significance was considered at P_adj_ ≤ 0.05. Relationships among production variables and DEMs were explored by Spearman correlations and significance were considered at *P* ≤ 0.05. Target genes of studied miRNAs and corresponding putative pathways were investigated using the miRWalk database (version 3.0) and Metacore™ software (release 6). Clustering was performed using the reads count for each animal of the DEMs using Permutmatrix software using Euclidean distance calculation and Ward’s minimum variance method [60]. Venn diagram was obtained using Venny 2.1 (https://bioinfogp.cnb.csic.es/tools/venny/index2.0.2.html).

## Additional file

Additional file 1: **Table S1.** Individual analysis of the expression of four miRNA in mammary gland. RT-qPCR analyses were performed from 11 cows (5 Holstein (H) and 6 Montbéliarde (M)). All miRNA levels were normalized to the values of 3 internal control miRNAs (*miR-26a, −92a, − 191*) and the results expressed as fold changes of threshold cycle (Ct) values relative to the control using the 2-ΔΔCt method. Results are presented as log_2_ ratio between M/H. TaqMan advance references are given for ech system. **Table S2.** Relationships among milk, fat, protein and lactose yields and 6 discussed differentially expressed miRNAs. The relationships were explored by Spearman correlations using 5 Holstein and 6 Montbéliarde cows. *** *P* < 0.001. ** *P* < 0.01. * *P* < 0.05. **Figure S1.** Comparison of percentage of match miRNA reads between libraries. Libraries were constructed from total RNA from mammary gland biopsies of 5 Holstein (H) and 6 Montbéliarde (M) cows. Blue correspond to percentage of unique-mapped, orange to multi-mapped and grey to unmapped reads. **Figure S2.** Correlation between Holstein and Montbéliarde miRNAs libraries. The correlation was calculated using log_2_ of the normalized counts of expressed miRNAs. **Figure S3.** Choice of 3 internal controls. Expression stability values (M) and rankings of the reference genes are determined by geNorm software. (DOCX 208 kb)

## Data Availability

The data supporting the conclusions of this article are within the paper and its additional files. The sequence data generated is publicly available in the NCBI GEO database with the accession GSE131057: (https://www.ncbi.nlm.nih.gov/geo/query/acc.cgi?acc=GSE131057)

## Abbreviations

DEMs: Differentially expressed miRNAs
EMT: Epithelial-to-mesenchymal transition
FC: Fold change
MG: Mammary gland
miRNA: MicroRNAs
nt: Nucleotides
