# Supplementary material for: Deep RNA-Seq reveals miRNome differences in mammary tissue of lactating Holstein and Montbéliarde cows
Source: BMC Genomics. 2019 Jul 30;20:621. doi: 10.1186/s12864-019-5987-4 (PMC6668132; doi:10.1186/s12864-019-5987-4)

**Additional file 1: Table S1**

**Table S2**

|  | **Milk** |  | **Fat yield** |  | **Protein yield** |  | **Lactose yield** |  |
| --- | --- | --- | --- | --- | --- | --- | --- | --- |
| ***miR 186*** | 0.86 | *** | 0.65 | * | 0.65 | * | 0.85 | ** |
| ***miR 30e 5p*** | 0.75 | ** | 0.68 | * | 0.63 | * | 0.71 | * |
| ***miR 16a*** | 0.75 | ** | 0.70 | * | 0.63 | * | 0.70 | * |
| ***miR 25*** | 0.04 | ns | 0.09 | ns | 0.05 | ns | -0.03 | ns |
| ***miR 100*** | -0.25 | ns | 0.00 | ns | -0.23 | ns | -0.33 | ns |
| ***miR 146b*** | -0.41 | ns | -0.08 | ns | -0.29 | ns | -0.44 | ns |

**Figure S1**


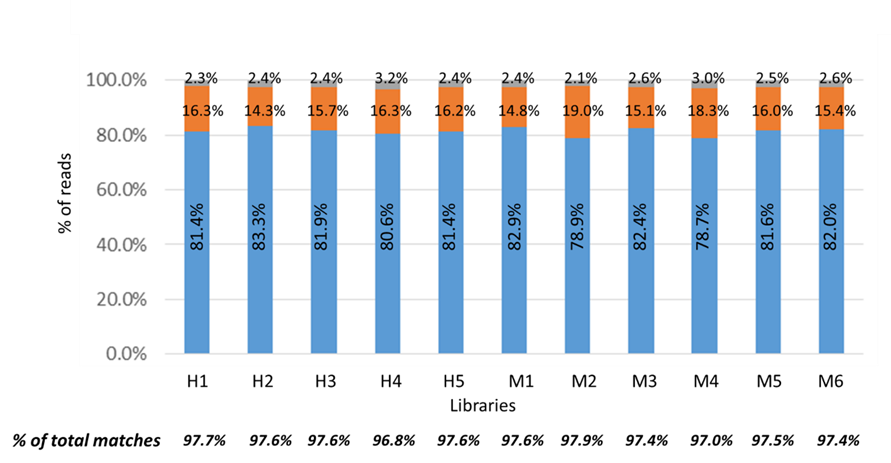


**Figure S2**

**Figure S3**


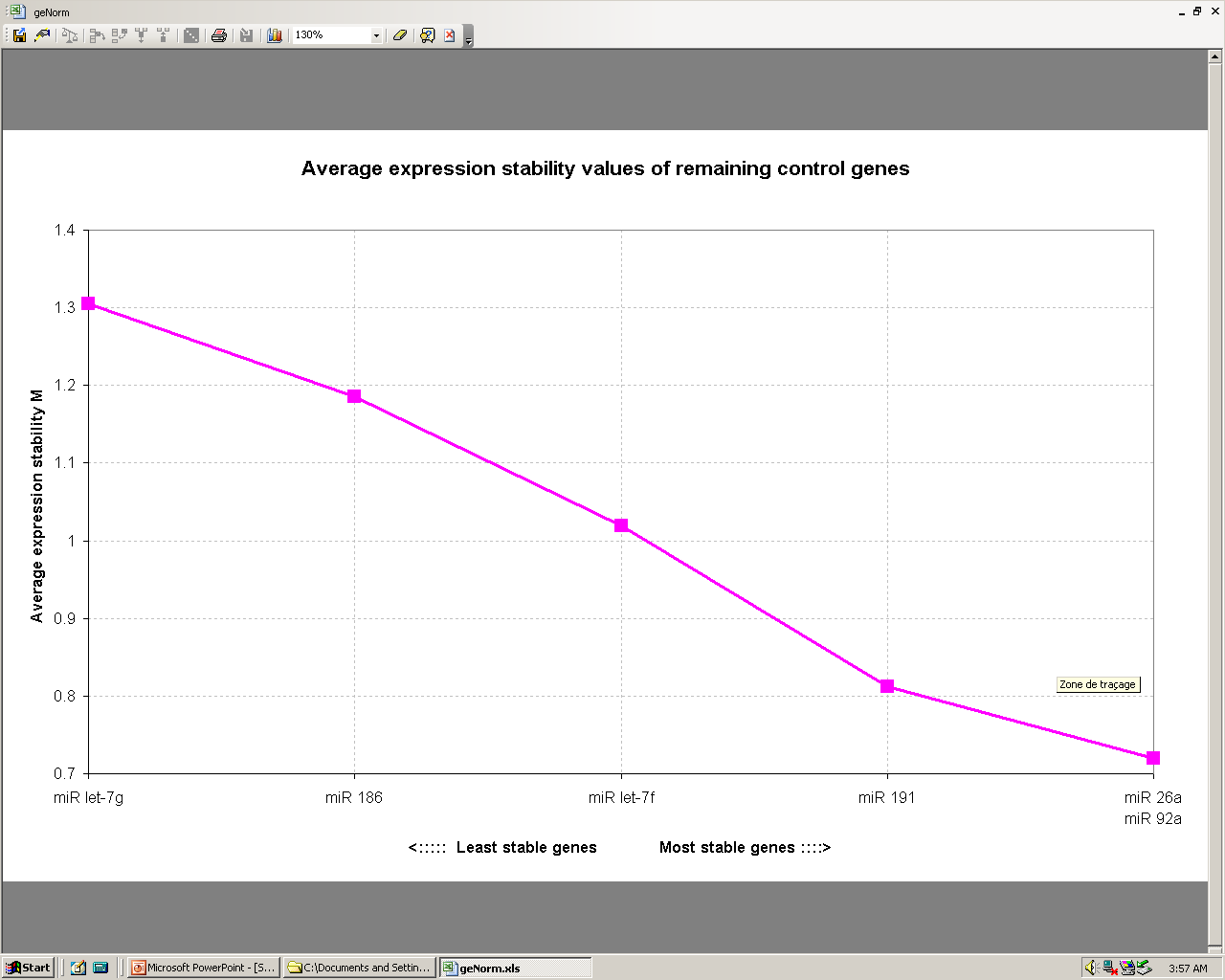

Supplement: Supplementary file 1 — Table S1. Individual analysis of the expression of four miRNA in mammary gland. RT-qPCR analyses were performed from 11 cows (5 Holstein (H) and 6 Montbéliarde (M)). All miRNA levels were normalized to the values of 3 internal control miRNAs (miR-26a, −92a, − 191) and the results expressed as fold changes of threshold cycle (Ct) values relative to the control using the 2-ΔΔCt method. Results are presented as log2 ratio between M/H. TaqMan advance references are given for ech system. Table S2. Relationships among milk, fat, protein and lactose yields and 6 discussed differentially expressed miRNAs. The relationships were explored by Spearman correlations using 5 Holstein and 6 Montbéliarde cows. *** P < 0.001. ** P < 0.01. * P < 0.05. Figure S1. Comparison of percentage of match miRNA reads between libraries. Libraries were constructed from total RNA from mammary gland biopsies of 5 Holstein (H) and 6 Montbéliarde (M) cows. Blue correspond to percentage of unique-mapped, orange to multi-mapped and grey to unmapped reads. Figure S2. Correlation between Holstein and Montbéliarde miRNAs libraries. The correlation was calculated using log2 of the normalized counts of expressed miRNAs. Figure S3. Choice of 3 internal controls. Expression stability values (M) and rankings of the reference genes are determined by geNorm software. (DOCX 208 kb) [file 12864_2019_5987_MOESM1_ESM.docx]
